# The Effect of Visual Feedback on Plaque Control: An RCT Comparing Toothbrushing Methods in Periodontitis Patients

**DOI:** 10.3390/jcm14020436

**Published:** 2025-01-11

**Authors:** Manon Tytgat, Maarten Glibert, Jeroen Callens, Sabine Lamoral, Paulien Van Gyseghem, Fien Decabooter, Véronique Christiaens

**Affiliations:** Oral Health Sciences Department of Periodontology and Oral Implantology, Ghent University, 9000 Ghent, Belgium; maarten.glibert@ugent.be (M.G.); vchristi.christiaens@ugent.be (V.C.)

**Keywords:** toothbrush, periodontitis, visual feedback

## Abstract

**Background/Objectives:** As gingivitis prevalence is closely related to plaque accumulation, effective oral hygiene is mandatory for maintaining healthy gingival tissues. The aim of this study was to evaluate the impact of different toothbrushing methods (a manual toothbrush (group 1 = MTB, the control); an electric toothbrush (group 2 = ETB); and an electric toothbrush with daily visual feedback (group 3 = ETBV)) on the plaque levels and periodontal health in patients after receiving initial periodontal treatment. **Methods**: A total of 67 patients were initially screened in this study, and 60 patients were randomly allocated into 1 of the 3 groups, with 53 patients completing this study. At baseline, the average probing depth across all groups was 3.60 mm, with an average bleeding on probing (BoP) score of 69,15%. Clinical parameters were assessed at multiple time points over 12 weeks. **Results**: The plaque levels reduced significantly in all groups: MTB decreased from 65,57% to 33.26%, ETB from 64,17% to 31.49%, and ETBV from 63,04% to 26.25% (*p* < 0.001 for all groups). Furthermore, a significant improvement for BoP was recorded across all groups: BoP decreased to 25.68% (MTB), 22.32% (ETB), and 23.14% (ETBV) (*p* < 0.001 for all groups). However, no significant difference in the plaque removal efficacy was observed between manual and electric toothbrushes, irrespective of visual feedback (*p* > 0.190). A linear mixed model analysis showed a significant overall group effect on the periodontal health parameters (*p* = 0.041) but no interaction with time (*p* = 0.965). **Conclusions**: These findings emphasize the importance of consistent oral hygiene in reducing periodontal disease. Further research is necessary to explore effective strategies for enhancing patient adherence and optimizing health outcomes.

## 1. Introduction

The widespread prevalence of gingivitis is primarily attributed to high levels of plaque accumulation. The critical role of plaque in the etiology of gingival inflammation is well described by Löe et al. [1]. The reinstatement of proper oral hygiene in cases of gingivitis often results in full recovery of the gingival tissues. In contrast to gingivitis, periodontitis is defined by the gradual destruction of the structures supporting the teeth. The diagnosis of periodontitis is based on clinical examination. Key diagnostic indicators include the presence of periodontal pockets, bleeding on probing (BoP), clinical attachment loss (CAL), and radiographic evidence of alveolar bone loss [2]. Although periodontitis is treatable, if left untreated, it can result in tooth loss. This condition is a chronic, multifactorial inflammatory disease linked to dysbiotic dental plaque biofilms [3].

Following the European Federation of Periodontology (EFP) guideline on the treatment of periodontal diseases, the initial phase focuses on encouraging behavioral changes. This phase aims to enhance patient motivation and improve skills in removing supragingival biofilm and managing risk factors. The subsequent phase, cause-related therapy, targets control or elimination of the subgingival biofilm and calculus through subgingival instrumentation [4].

Research conducted over the past decade [5,6,7,8,9,10,11] has consistently shown that specific powered toothbrushes, such as oscillating–rotating models, are effective in plaque removal and reducing gingival inflammation [12,13,14]. Despite the known benefits of mechanical cleaning in controlling supragingival plaque, maintaining consistent oral hygiene remains challenging for many individuals [15]. However, electric toothbrushes have been found to be more effective in helping patients maintain optimal oral hygiene routines, offering a reliable alternative [16,17,18]. As noted by Warren et al. [19], electric toothbrushes are a valuable component of daily oral care due to their higher efficacy and improved patient compliance.

The integration of visual feedback through smartphone applications has emerged as a promising approach to enhancing oral hygiene practices. These applications, when paired with smart toothbrushes, provide real-time guidance and personalized feedback, enabling users to monitor and improve their brushing techniques. By utilizing sensors and artificial intelligence, these tools track brushing duration, coverage, and pressure. They offer insights that encourage better oral care habits. Studies have shown that such interactive technologies can lead to significant improvements in plaque control and gingival health [20,21,22], though these results were not statistically significant [23]. Notably, Gurnani et al. (2023) demonstrated that a mobile app significantly enhanced oral health in children with ADHD, highlighting the potential of digital interventions for this specific population [24].

Despite the proven effectiveness of electric toothbrushes, many studies on their impact have been short-term and have not specifically addressed periodontitis patients, who require more intensive oral hygiene. This study aims to evaluate whether an electric toothbrush with visual feedback offers additional benefits in reducing plaque and improving gingival health compared to a standard electric toothbrush in periodontitis patients. The focus was on a 12-week period to assess the effects of these toothbrushes following non-surgical periodontal treatment.

The null hypothesis is that there would be no significant difference in plaque reduction or gingival health improvements between the different brushing groups, with and without visual feedback.

## 2. Materials and Methods

### 2.1. Patient Selection

Patients diagnosed with periodontitis Class II or III according to the EFP criteria were enrolled between February 2022 and April 2024 to participate in this multi-center RCT comparing the use of 3 different toothbrushes. The centers of inclusion were the Department of Periodontology & Oral Implantology at Ghent University Hospital and 2 private practices. Patients were selected based on the inclusion and exclusion criteria.

### 2.2. Inclusion Criteria

The inclusion criteria were as follows:

- ≥18 years of age;
- No systemic diseases;
- Minimum of 5 evaluable teeth in each quadrant;
- Periodontitis stage II or III according to the EFP criteria.

### 2.3. Exclusion Criteria

The exclusion criteria were as follows:

- Pregnancy;
- Smoking;
- Systemic diseases;
- Adverse medical history or long-term medication use;
- Patients with partial removable dentures;
- Active orthodontic treatment and/or orthodontic wire.

This study was ethically approved by the Ethical Committee of Ghent University Hospital (BC-11037) on 10 February 2022 and registered at ClinicalTrials.gov (NCT05723133). It was conducted in accordance with the ethical principles of the Declaration of Helsinki in 1975, as revised in 2013 [25]. Informed consent was obtained from all participants.

### 2.4. Sample Size Determination

This randomized controlled trial (RCT) was designed to detect differences in bleeding on probing (BoP) with 80% power and a 5% significance level, based on an a priori power analysis using a one-way ANOVA. The sample size calculation followed the methodology outlined in Schmalz et al. (2017), which determined that 18 participants per group would provide an actual power of 0.838 with an effect size of 0.85. To account for an anticipated 10% dropout rate, 20 participants per group were enrolled [26].

### 2.5. Study Setup

The present study was a randomized controlled trial with an examiner-masked, three-group, parallel design consisting of five consultations: (visit 1) intake followed by initial non-surgical periodontal therapy, oral hygiene follow-up at 3, 6, and 9 weeks after initial therapy (=visits 2, 3, and 4), and a periodontal reassessment at 12 weeks (visit 5). Three experience periodontitis working in 3 different periodontal practices (Ghent University Hospital and 2 private practices) in Belgium were selected to collaborate in this multi-center RCT.

### 2.6. Randomization

Patients were randomly allocated with closed envelopes, using the block randomization “AABBCC”, into either the control group (manual toothbrush = MTB) or one of the 2 test groups: an electric toothbrush without daily visual feedback (=ETB) or an electric toothbrush with daily visual feedback (=ETBV).

In the last group (=ETBV), visual feedback was given by the Oral-B application using artificial intelligence for guidance in a 2 min brushing session with 3D teeth tracking via the Oral-B iO app with Bluetooth connectivity. All of the participants in the ETB and ETBV groups received an Oral-B iO Series 9N (Braun GmbH, Kronberg, Germany), while the participants in the MTB group received an Oral-B 1.2.3. indicator toothbrush (Procter & Gamble, Newbridge, Ireland).

### 2.7. Non-Surgical Periodontal Treatment

The initial non-surgical periodontal treatment included supra- and subgingival cleaning with oral hygiene instructions to obtain optimal periodontal health. Participants were instructed to use either a manual or electric toothbrush (as per the group allocation) for the following 12 weeks. All of the patients used the same toothpaste (Oral-B Pro Expert, Procter & Gamble, Manufacturing Ireland Ltd., Newbridge, Co. Kildare, Ireland).

At the start of this study, as well as during the follow-up visits, the participants received personal brushing instructions, focusing on both the proper toothbrush technique and the use of correctly sized interdental brushes to ensure effective oral hygiene. Three weeks after the initial treatment, patients were scheduled in for their first follow-up (= visit 2). During this consultation, their oral hygiene was evaluated, and the oral hygiene instructions were repeated. Visits 3 and 4, respectively, 6 and 9 weeks after the initial treatment, were identical to visit 2. A final periodontal reassessment occurred at visit 5 (12 weeks).

The primary outcome variable was pocket probing depth (PPD), which was used as the main indicator of periodontal health and treatment effectiveness in this study. PPD was assessed at the beginning (visit 1) and end of this study (visit 5). Secondary outcome variables were investigated to provide a broader understanding of the possible impact of varied toothbrushes. The secondary outcome variables were bleeding on probing (BoP), plaque levels (PI), and the percentage of sites with pocket depths greater than or equal to 4 mm, greater than or equal to 6 mm, and greater than or equal to 8 mm.

### 2.8. Clinical Examination and Indices

The clinical examination included a periodontal chart, taken at intake (visit 1) and 12 weeks after the initial treatment (visit 5). The following parameters were included: present teeth, teeth replaced by implants, pocket probing dept (PPD), bleeding on probing (BoP), and the presence of plaque (PI).

Bleeding on probing was assessed using a dichotomous score, the gingival margin was probed at ∼60° to the longitudinal axis of the tooth, and the absence (0) or presence (1) of bleeding was scored within 30 s. For the PI, 6 surfaces were examined per tooth (disto-buccal, mid-buccal, mesio-buccal, disto-lingual, mid-lingual, and mesio-lingual). Additionally, intra-oral clinical pictures were taken after applying a plaque detector (GC Tri Plaque ID Gel, GC Europe NV, Leuven, Belgium). BoP and PI were measured and clinical pictures were collected during every study visit.

All clinical examinations were performed by the same experienced examiners (J.C., P.V.G., F.D., and M.T.). The examiners were masked to the group allocation, and records from prior visits were not available at the time of reexamination.

### 2.9. Statistical Analysis

The data were analyzed using SPSS (version 29, IBM Corp., Armonk, NY, USA). Descriptive statistics were used to summarize the data, including means and percentages for PPD, BoP, PI, and CAL. Comparisons between the three treatment groups (MTB, ETB, and ETBV) for PPD, BoP, and PI were conducted using a linear mixed model approach, which accounted for fixed effects (group assignment, time, and their interactions) and random effects (individual variability). This model allowed for an assessment of the impact of each treatment on the periodontal outcomes over time.

## 3. Results

### 3.1. Patients

**Figure 1:**
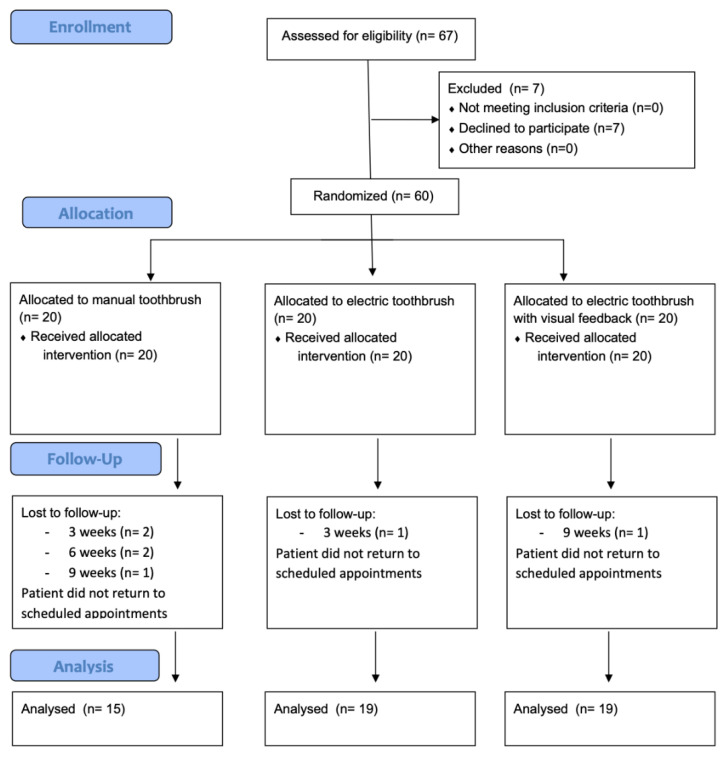
Flowchart of CONSORT diagram 2010.

A total of 67 participants were initially recruited for this study. A total of 7 individuals chose not to participate, and the remaining 60 participants were allocated across the three study groups. In total, 53 participants successfully completed the 12-week study protocol. See Figure 1.

### 3.2. Primary and Secondary Outcomes

At baseline (see Table 1 and Table 2), the mean age of the participants was 53.98 years (95% CI = 49.62–58.34; range = 22–82). Their average number of teeth was 25.88 (95% CI = 24.88–26.88; range = 16–32), and the mean PPD was 3.77 mm (95% CI = 3.60–3.94; SD = 0.62), the mean BoP was 69.27% (95% CI = 62.59–75.96; SD = 23.76), and the mean PI was 64.14% (95% CI = 56.80–71.48; SD = 26.09).

At intake, the average BoP varied by group: MTB = 71.81%, ETB = 66.92%, and ETBV = 69.14% (*p* = 0.041). During the final study consultation, significant reductions in the average BoP were observed: MTB = 25.68% (*p* < 0.001), ETB = 22.32% (*p* < 0.001), and ETBV = 23.14% (*p* < 0.001). The linear mixed model indicated a significant overall group effect (*p* = 0.041) but no significant interaction with week (*p* = 0.965).

The plaque scores at baseline were MTB = 65.57%, ETB = 64.17%, and ETBV = 63.04% (*p* = 0.190). By week 12, these scores significantly decreased: MTB = 33.26% (*p* < 0.001), ETB = 31.49% (*p* < 0.001), and ETBV = 26.25% (*p* < 0.001). The linear mixed model showed no significant group differences (*p* = 0.190) or interactions (*p* = 0.917).

For the average pocket depths, the baseline measurements were MTB = 3.62 (SD = 0.45), ETB = 3.90 (SD = 0.44), and ETBV = 3.74 (SD = 0.44) (*p* = 0.412). By week 12, these values significantly decreased: MTB = 2.99 (SD = 0.45) (*p* < 0.001), ETB = 2.81 (SD = 0.44) (*p* < 0.001), and ETBV = 2.90 (SD = 0.44) (*p* < 0.001). The overall reduction in the pocket depths was significant (*p* < 0.001), with no significant group effect (*p* = 0.940) or interaction between group and week (*p* = 0.269).

For the pocket depths, the baseline percentages for a PD ≥ 4 mm were MTB = 40.61%, ETB = 55.68%, and ETBV = 45.98% (*p* = 0.412). By week 12, these values significantly decreased: MTB = 21.81 (*p* < 0.001), ETB = 16.11% (*p* < 0.001), and ETBV = 19.66% (*p* < 0.001). The overall reduction was significant (*p* < 0.001), with no significant group effect (*p* = 0.549) or interaction (*p* = 0.056).

For pocket depths of ≥ 6 mm, the baseline values were MTB = 10.01%, ETB = 12.58%, and ETBV = 11.08% (*p* = 0.678). By week 12, MTB decreased to 3.35% (*p* = 0.002), ETB to 3.14% (*p* = 0.003), and ETBV to 2.75% (*p* = 0.001). The linear mixed model revealed no significant group effect (*p* = 0.808) or interaction (*p* = 0.776).

Lastly, for pocket depths of ≥ 8 mm, the baseline percentages were MTB = 1.66%, ETB = 2.14%, and ETBV = 2.20% (*p* = 0.841). By week 12, these values were reduced to MTB = 0.37% (*p* = 0.030), ETB = 0.80% (*p* = 0.045), and ETBV = 0.92% (*p* = 0.042). The analysis showed no significant group effects (*p* = 0.747) or interactions (*p* = 0.999).

**Figure 2:**
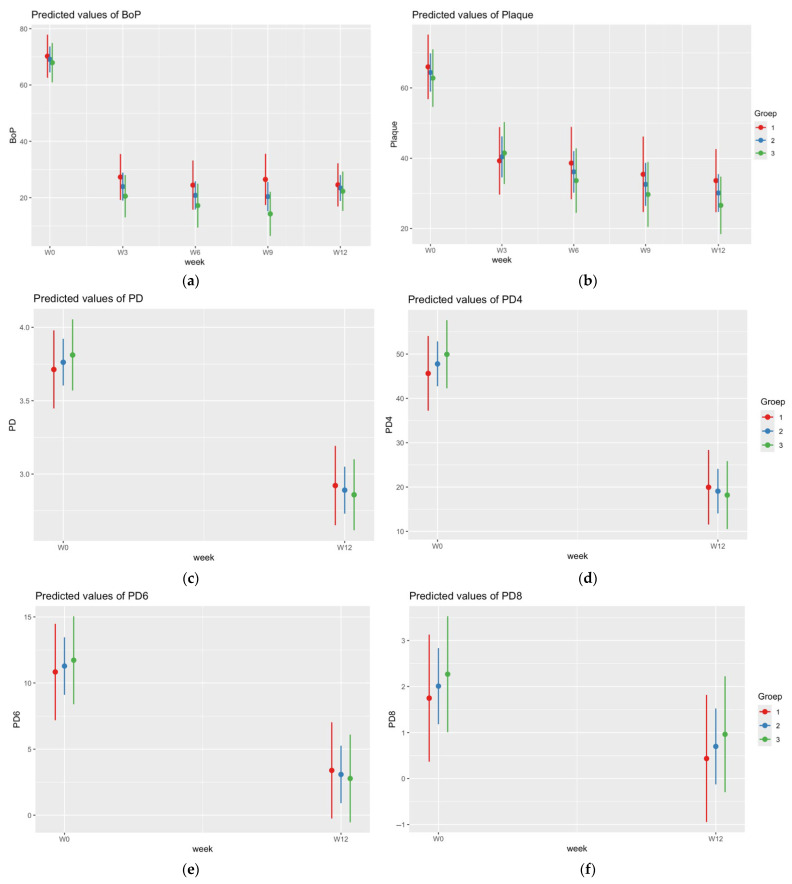
Predicted values of BoP (**a**), plaque (**b**), PD (**c**), PD ≥ 4 mm (**d**), PD ≥ 6 mm (**e**), and PD ≥ 8 mm (**f**).

The predicted values for BoP, plaque, PD, a PD ≥ 4 mm, a PD ≥ 6 mm, and a PD ≥ 8 mm are also illustrated in Figure 2.

## 4. Discussion

While proper daily oral hygiene is essential for maintaining gingival health, many individuals face challenges in sustaining optimal gum health over extended periods. Multiple studies concluded that the plaque removal efficacy of electric toothbrushes has significantly improved, and they are recognized by both dental professionals and patients as effective alternatives to manual brushing [8].

This study shows significant improvements in the periodontal health indicators over the 12-week intervention period, with reductions in the bleeding on probing (BoP), plaque levels, and pocket depths across all groups. While these changes are statistically significant, the clinical relevance may be limited. The lack of significant differences between the groups, despite controlled conditions such as frequent follow-ups (every 3 weeks) and strict supervision, suggests that consistent oral hygiene may be more important than the type of toothbrush used. These results align with the existing literature on the importance of regular dental care and effective brushing techniques in managing periodontal health.

The significant reduction in bleeding on probing, from an average of 69,15% at baseline to as low as 22.32% by week 12, indicates a marked decrease in gingival inflammation. This decline was observed across all groups, though group 1 exhibited the highest initial levels of inflammation and a relatively slower reduction in bleeding on probing compared to groups 2 and 3. Supporting this, Yacoob et al. (2014) found that powered toothbrushes are more effective than manual toothbrushes in reducing plaque and gingivitis over both short- and long-term periods. Their study revealed an 11% reduction in plaque, as measured by the Quigley–Hein (Turesky) index, within one to three months and a 21% reduction in plaque after more than three months of use [11].

Previous research on toothbrushes has focused mainly on the reversal of gingivitis and the effects of different oral hygiene practices on inflammation. However, there are limited data on the role of visual feedback in electric toothbrushes and its potential impact on periodontal treatment. Comparing our findings with those of Erbe et al. (2019) and Grender et al. (2022), both of which focused on plaque reduction, the present study also included gingival bleeding as a key marker of periodontal health. Erbe et al. conducted a 6-week trial and reported a greater plaque reduction with an interactive electric toothbrush, with TMQHPI changes of 0.777 and 0.834 at 2 and 6 weeks, respectively. Grender et al. observed a superior plaque reduction with an electric toothbrush over 12 weeks, particularly in the gingival margin and interproximal regions. In contrast, our study found significant plaque reductions across all groups but no significant differences between manual and electric toothbrushes, even with visual feedback. This suggests that while advanced brushing technologies may offer short-term benefits, consistent oral hygiene practices are crucial for long-term improvements. Grender et al. also noted a higher percentage of participants transitioning from a “not healthy” to a “healthy” gingival status with the electric toothbrush, an effect observed in our study as well, although the magnitude of the change was similar across all groups. Thus, sustained oral hygiene, rather than technology alone, appears to be key to meaningful improvements in both plaque control and gingival health over time [6,27].

The discrepancy between our findings (MTB = 33.26%, ETB = 31.49%, and ETBV = 26.25%) and those of Erbe et al. (2018), where interactive electric toothbrushes resulted in a 34% reduction in plaque scores compared to only 1.7% with manual brushing, could be attributed to the different populations and study conditions. Erbe et al.’s adolescent cohort may have responded more strongly to the interactive feedback, or novelty effects may have influenced their results, which was not observed in this study [28].

This study has several key strengths. Its randomized controlled trial (RCT) design minimizes selection bias and enhances its internal validity. The multi-center approach broadens the generalizability of the findings across different settings. Additionally, blinding of the examiners reduced observer bias, ensuring accurate assessments of the periodontal outcomes. The longitudinal design with frequent follow-ups (every 3 weeks) allowed for a thorough evaluation of the interventions over time. Finally, this study’s robust statistical analysis strengthens the reliability of its conclusions by controlling for confounders and handling missing data appropriately.

Several limitations of this study should also be considered. The controlled environment with strict follow-ups may not reflect real-world conditions, and this monitoring may have influenced participant compliance, reducing the likelihood of detecting significant group differences. Moreover, the relatively short follow-up period may not have been sufficient to capture long-term differences in plaque and gingival health, and high compliance could have led to performance bias, as participants might have adhered more strictly to oral hygiene routines than they would in daily life. As noted by Yacoob et al. (2014), the benefits of powered toothbrushes increase over time, suggesting that a longer follow-up might have yielded more pronounced differences in plaque control [6,11,27,29].

In addition, there was no control over the brushing technique or frequency, and no data were available regarding these factors. While we attempted to standardize the participant behavior with detailed and standardized instructions, individual variations in technique or frequency could have influenced the results. The absence of precise control in this area may have introduced variability into the data that could have confounded the outcomes.

The higher dropout rate in the manual toothbrush group (71.43% of all dropouts) may have skewed the results further. The final sample size may have been insufficient to detect more subtle differences, especially given the higher-than-expected dropout rate. Additionally, the use of a dichotomous plaque assessment tool with stringent criteria may have oversimplified plaque measurement, potentially overlooking subtle differences between groups. Future studies could benefit from more sophisticated methods, such as artificial intelligence (AI)-enhanced plaque evaluation, which could provide more detailed insights into oral hygiene outcomes.

The cost-effectiveness of powered toothbrushes, particularly those with features like visual feedback, is another important consideration. It would have been valuable to assess whether participants were willing to pay extra for these features, which could help determine their real-world value. Understanding patient preferences regarding toothbrush types and features would also provide insights into long-term compliance and satisfaction. Surveying participants after this study on their preferences could have yielded useful data for tailoring oral care recommendations to individual needs.

Lastly, the visual feedback feature of electric toothbrushes may not be as accessible to older individuals or those less familiar with technology. This limitation could reduce the applicability of such devices for certain populations, especially given the higher prevalence of periodontal disease among older adults. Future research might also explore the broader health implications of oral care, particularly its links to systemic diseases such as cardiovascular disease, diabetes, Parkinson’s, and depression, as well as its impact on specific patient groups with disabilities or behavioral challenges [24,30].

## 5. Conclusions

In conclusion, this study reinforces the critical role of effective oral hygiene interventions in improving periodontal health. The significant reductions in PPD, BoP, and PI across all groups suggest that regular dental care and patient education are vital components in managing periodontal disease. Importantly, no significant differences were observed between the groups, highlighting that consistent oral hygiene practices are more influential than technological advancements in achieving positive clinical outcomes. These findings underscore the importance of patient education and adherence to daily oral hygiene routines.

For dental professionals, this study provides valuable insight into the continued emphasis on foundational care in periodontal disease management. By focusing on effective and consistent oral hygiene practices, clinicians can help optimize patient outcomes, regardless of the specific technologies employed.

## Figures and Tables

**Table 1:** Baseline characteristics for groups MTB, ETB, and ETBV.

	MTB	ETB	ETBV
Age	54 (SD = 16)	55 (SD = 14)	52 (SD = 16)
Teeth	26 (SD = 3)	25 (SD = 3)	27 (SD = 4)

TB = manual toothbrush; ETB = electric toothbrush; ETBV = electric toothbrush with visual feedback.

**Table 2:** Clinical parameters for MTB, ETB, and ETBV at different time points.

	Group	W0 (Mean ± SD)	W3 (Mean ± SD)	W6 (Mean ± SD)	W9 (Mean ± SD)	W12 (Mean ± SD)	*p*-Value (Group × Time Interaction)
**(PD) (mm)**	MTB	3.62 ± 0.45	3.71 ± 0.45	3.42 ± 0.43	3.15 ± 0.44	2.99 ± 0.45	0.940
	ETB	3.90 ± 0.72	3.88 ± 0.72	3.65 ± 0.72	3.15 ± 0.50	2.81 ± 0.44	
	ETBV	3.74 ± 0.52	3.80 ± 0.55	3.55 ± 0.51	3.12 ± 0.47	2.90 ± 0.47	
**BoP (%)**	MTB	71.81 ± 22.43	66.50 ± 21.83	54.35 ± 19.54	46.68 ± 18.27	25.68 ± 15.64	0.041
	ETB	66.92 ± 27.00	62.75 ± 25.24	50.88 ± 23.47	42.32 ± 20.98	22.32 ± 15.35	
	ETBV	69.14 ± 22.05	64.10 ± 20.15	52.78 ± 21.58	44.45 ± 19.35	23.14 ± 13.68	
**Plaque Index (%)**	MTB	65.57 ± 27.08	61.25 ± 26.67	57.12 ± 25.47	48.50 ± 21.87	33.26 ± 15.49	0.190
	ETB	64.17 ± 22.45	61.21 ± 20.38	57.02 ± 22.01	46.88 ± 20.03	31.49 ± 14.36	
	ETBV	63.04 ± 30.10	59.90 ± 27.91	55.11 ± 27.47	45.67 ± 24.88	26.25 ± 15.73	
**PD ≥ 4 mm (%)**	MTB	40.61 ± 20.32	39.50 ± 18.78	36.50 ± 19.00	33.33 ± 18.50	21.81 ± 16.85	0.549
	ETB	55.68 ± 20.46	52.25 ± 21.35	48.65 ± 20.17	42.13 ± 19.72	16.11 ± 15.59	
	ETBV	45.98 ± 18.97	43.40 ± 17.30	41.15 ± 17.85	36.22 ± 16.24	19.66 ± 14.68	
**PD ≥ 6 mm (%)**	MTB	10.01 ± 9.62	8.50 ± 7.80	7.28 ± 6.72	6.10 ± 5.24	3.35 ± 5.17	0.808
	ETB	12.58 ± 12.90	11.25 ± 11.55	10.25 ± 11.45	8.12 ± 10.60	3.14 ± 6.11	
	ETBV	11.08 ± 6.82	9.50 ± 5.91	8.60 ± 6.25	7.28 ± 5.17	2.75 ± 3.06	
**PD ≥ 8 mm (%)**	MTB	1.66 ± 2.69	1.50 ± 2.40	1.45 ± 2.12	1.20 ± 2.10	0.37 ± 0.87	0.747
	ETB	2.14 ± 5.16	1.80 ± 4.76	1.65 ± 4.43	1.20 ± 3.72	0.80 ± 2.59	
	ETBV	2.20 ± 3.01	2.00 ± 2.50	1.85 ± 2.70	1.60 ± 2.10	0.92 ± 1.61	

MTB = manual toothbrush; ETB = electric toothbrush; ETBV = electric toothbrush with visual feedback; PD = probing depth; BoP = bleeding on probing.

## Data Availability

Due to privacy restrictions, the supporting data are not publicly available. However, data can be requested from the corresponding author.
